# Mathematical Modeling Evaluates How Vaccinations Affected the Course of COVID-19 Disease Progression

**DOI:** 10.3390/vaccines11040722

**Published:** 2023-03-24

**Authors:** Eleftheria Tzamali, Vangelis Sakkalis, Georgios Tzedakis, Emmanouil G. Spanakis, Nikos Tzanakis

**Affiliations:** 1 Computational Biomedicine Laboratory, Institute of Computer Science, Foundation for Research and Technology-Hellas (FORTH), 70013 Heraklion, Greece; 2 Department of Respiratory Medicine, University Hospital of Heraklion, Medical School, University of Crete, 71003 Heraklion, Greece

**Keywords:** SARS-CoV-2, COVID-19, mathematical modeling, vaccination, epidemics, scenario analyses, SEIR model

## Abstract

The regulation policies implemented, the characteristics of vaccines, and the evolution of the virus continue to play a significant role in the progression of the SARS-CoV-2 pandemic. Numerous research articles have proposed using mathematical models to predict the outcomes of different scenarios, with the aim of improving awareness and informing policy-making. In this work, we propose an expansion to the classical SEIR epidemiological model that is designed to fit the complex epidemiological data of COVID-19. The model includes compartments for vaccinated, asymptomatic, hospitalized, and deceased individuals, splitting the population into two branches based on the severity of progression. In order to investigate the impact of the vaccination program on the spread of COVID-19 in Greece, this study takes into account the realistic vaccination program implemented in Greece, which includes various vaccination rates, different dosages, and the administration of booster shots. It also examines for the first time policy scenarios at crucial time-intervention points for Greece. In particular, we explore how alterations in the vaccination rate, immunity loss, and relaxation of measures regarding the vaccinated individuals affect the dynamics of COVID-19 spread. The modeling parameters revealed an alarming increase in the death rate during the dominance of the delta variant and before the initiation of the booster shot program in Greece. The existing probability of vaccinated people becoming infected and transmitting the virus sets them as catalytic players in COVID-19 progression. Overall, the modeling observations showcase how the criticism of different intervention measures, the vaccination program, and the virus evolution has been present throughout the various stages of the pandemic. As long as immunity declines, new variants emerge, and vaccine protection in reducing transmission remains incompetent; monitoring the complex vaccine and virus evolution is critical to respond proactively in the future.

## 1. Introduction

On 11 March 2020, the World Health Organization declared coronavirus disease (COVID-19) as a pandemic, caused by a coronavirus strain named SARS-coronavirus-2 (SARS-CoV-2). Three years after its initial outbreak, SARS-CoV-2 has evolved into milder strains yet still remains active, widespread, and a healthcare challenge due to its high transmission rates, immune evasion, and the complexity of its treatment. The virus mainly affects the human respiratory system but also affects the gastrointestinal tract, kidney system, liver, pancreas, eyes, and brain [1].

**Figure 1:**
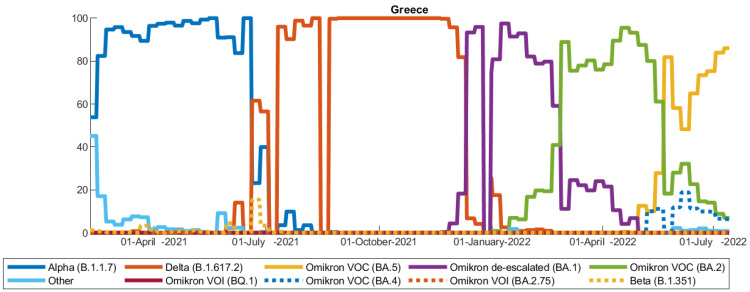
Proportion of SARS-CoV-2 variants in Greece for the time period 1 February 2021–15 June 2022. Data are provided by https://www.ecdc.europa.eu/ (accessed on 14 February 2023).

The highly dynamic and fast changing virus spread and evolution has caused multiple subsequent waves worldwide with varying regulation policies applied in an attempt to decrease the human losses and the socio-economic burden. We have witnessed three main phases; the first one was related to the initial wild-type strain—early 2020 until June 2020—at a time when the only possible mitigation action was to implement strict distancing and isolation of the population to minimize the explosion of the virus. The second one—from July 2020 until early December 2021—was the phase characterized by the appearance and dominance of several variants of SARS-CoV-2, while vaccination programs were activated worldwide, and finally, the third phase starting from January 2022 until today is the phase in which a high percentage of the population worldwide is fully vaccinated (apart from Africa), and yet new variants of SARS-CoV-2 are continuously emerging. Variants of interest (VOIs) that circulate widely are closely monitored and may become variants of concern (VOCs). The delta and omicron variants, for example, were of major concern due to their high transmissibility and immune escape capabilities which affected vaccinated people and recovered patients. Different lineages of omicron were identified soon after the emergence of omicron, as well as recombinant SARS-CoV-2 lineages derived from variants of omicron and delta proving the continuous evolution of the virus [2]. The proportion of SARS-CoV-2 variants over time in Greece is depicted in Figure 1.

Undoubtedly, COVID-19 vaccines [3,4] have proven to be highly effective against severe illness [5,6,7], even for the latest omicron variants [8]. However, the exact effectiveness of vaccines (in terms of infection, reinfection, and transmission) is still a vague parameter and highly dependent on virus variant’s characteristics [9,10,11,12]. Moreover, the immunity protection after vaccination or infection progressively declines with time, which has resulted in a necessary program of vaccine booster shots [13,14,15], whereas the escaping capabilities of the emergent variants to acquired immunity remain an open issue. Overall, the challenges we continuously face for COVID-19 include the virus evolution, the waning and escaping immunity, and the compromised vaccine protection at reducing transmission, along with the existing problem of vaccine inequity [16] affecting not only local communities but people worldwide.

In order to act proactively, it is necessary to be able to accurately simulate the spread dynamics and project the effect of vaccinations, booster shots programs, and control measures to inform, guide, and protect policy-making during the various phases and challenges of the pandemic. The insights gained from modeling the pandemic will equip us to better tackle comparable threats that may arise in the future.

Several research studies have employed mathematical models to investigate the consequences of different scenarios for decision-making in various medical domains, including both non-communicable diseases like cancer [17,18] and communicable ones. Specifically, for communicable diseases, these models can be broadly classified into three types: statistical, empirical, and state-space models. State-space models include various techniques, such as complex network modelling, agent-based simulations, stochastic Markov chain models, stochastic SEIR (susceptible, exposed, infectious, recovered), and deterministic SEIR models. These models have also been extensively used in the past for monitoring the progression of MERS and SARS diseases, with exceptional output and results [19]. Extensive research has been conducted on the impact of control measures and vaccinations to analyze their effect on COVID-19 [20,21,22,23,24,25,26,27,28,29,30,31]. Numerous studies that have focused on the early stages of vaccination programs have highlighted the need to avoid treating the vaccinated population as passive bystanders. These studies have suggested that strict regulation measures should be maintained until a significant proportion of the population is vaccinated [24,28,29]. In another work, a SEIR model was used to account for the observation that asymptomatic individuals can infect susceptible individuals. The authors explored various scenarios, including the impact of non-pharmaceutical interventions (such as social distancing, self-isolation, and contact-tracing) and pharmaceutical interventions (such as testing and mass vaccination). They also considered the possibility of losing acquired immunity over time and reinfection [32]. The study of epidemics, disease spreading, and optimal immunization has been also investigated in view of the percolation theory in graphs. Targeted immunization has been studied in networks with limited knowledge (only a small portion of the whole network structure is usually known) and temporary immunity (a node that acquires immunity at one step may lose it later). Interestingly, the authors concluded that increasing the knowledge level in targeted immunization may not be as effective as one might expect when dealing with diseases such as COVID-19. In a work closer to ours, Parolini et al. [33] proposed a new epidemiological compartmental model accounting for new variants (i.e., the delta variant) and vaccination in order to better identify specific epidemic trends (such as new outbreaks associated with the growth of a more transmissible virus variant). In their scenario analysis, they investigated whether the Green Pass strategy at schools and universities, which allowed access strictly to vaccinated individuals, could produce a new epidemic wave. The main aim of their work was to improve the forecast quality. There are a limited number of studies that examine the vaccination period within a realistic vaccination program setting, addressing important questions such as the impact of vaccines in protecting the population and the measures needed to control the spread of the disease through vaccination. Our research is dedicated to this particular period and involves monitoring the epidemiological trends as new variants, such as delta and omicron, emerge and spread.

In our work, an expansion to the classical SEIR epidemic model is proposed, aiming to fit the complex epidemiological data of COVID-19 in Greece. The proposed model includes additional compartments for vaccinated, asymptomatic, hospitalized, and deceased individuals, splitting the population into two branches based on the severity of progression. By incorporating the realistic vaccination program implemented in Greece into the model, this study provides a more accurate and comprehensive understanding of how policy decisions affect the spread of COVID-19. This study is the first to investigate how alterations in the responsiveness of citizens to the vaccination program, the vaccine effectiveness to infection and onward transmission, and the time frame within which the acquired immunity provides protection affect the dynamics of COVID-19 spread (incidents, hospitalizations, mortality) in Greece at crucial time-intervention points. Furthermore, the proposed model allows specific transition rates to be time-varying and data-calibrated to enable them to express the potential role of vaccinations and virus evolution on hospitalizations and mortality.

## 2. Methods

### 2.1. Adapted and Modified SEIR Model

**Figure 2:**
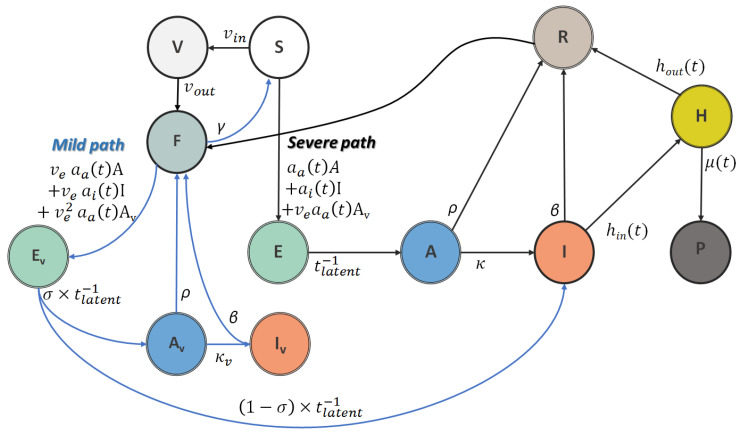
Schematic overview of the proposed model (SEAIR-severe/mild). Individuals in the model are classified into susceptible (S), unvaccinated exposed (E), unvaccinated asymptomatic (A), unvaccinated detected incidents (I), recovered (R), hospitalized (H), perished (P), vaccinated before immunity has been reached (V), fully vaccinated (F), vaccinated exposed (Ev), vaccinated asymptomatic (Av) and vaccinated detected incidents (Iv).

In this work, COVID-19 spread dynamics are described through adaptations of a SEIR compartmental model [30,34]. In this broadly used framework, the whole population is divided into homogeneous (mean-field approximation) groups, usually called compartments. Individuals transit between compartments, but can only be in one compartment at a given time point. In SEIR models, the whole population N is divided into: (i) a susceptible population (S), (ii) exposed individuals (E), which have been infected but are not yet infectious (considering a latency period), (iii) infected people (I), and (iv) recovered or removed people (R), which correspond to people that have either recovered or perish from the disease. Our research involves dividing the entire population into two main branches resembling the SEIR model. The first branch depicts a potentially more severe progression of the disease, while the second branch portrays a milder progression. In short, our study involves categorizing individuals as pure susceptibles, denoted by the compartment S, who are at risk of contracting the disease from both branches. Depending on the vaccination rate, the susceptible population progressively becomes vaccinated and follows the milder progression path. These individuals can also become infected, yet with milder symptoms, and can also spread the disease. Assuming a certain vaccine inefficacy to severe illness, some of these individuals may follow the more severe branch. A schematic illustration of the proposed model is shown in Figure 2. In more detail, we assume that the infected population is composed of those individuals that have been confirmed as COVID-19 patients (I) and those that are asymptomatic (A) and remain undetected in the system. Furthermore, we distinguish between the recovered individuals (R) and those that perish from the disease (P). In addition, we assume that confirmed patients can either recover at home (mild incidents) or be hospitalized (severe incidents). Furthermore, we take into account the vaccinated individuals including two additional compartments: the vaccinated (V) and the immune (F). The vaccinated individuals are those who have completed the initial vaccination protocol (a second dose of a two-dose vaccine such as the Pfizer/BioNTech and Moderna vaccine, or a first dose of a one-dose vaccine such as the Johnson and Johnson vaccine). Vaccinated people are considered immune after a period of time, where the human body reaches a threshold of protection. In other words, the immune population is the vaccinated population after a certain period of time. The booster shot (a third dose of the Pfizer/BioNTech vaccine, or a second dose of the Johnson and Johnson vaccine) is also accommodated in the model. In that case, susceptible individuals are directly transferred to the immune population with a rate equal to the vaccination rate of the third dose. The vaccinated (and recovered) individuals are susceptible to infection, yet progress to the mild path instead, as exposed (Ev) and asymptomatic (Av) individuals. The vaccinated confirmed cases (Iv) are not considered in the current version due to a lack of related data. Furthermore, recovered and vaccinated individuals can become susceptible to severe illness again due to a loss of immunity.

The model is described mathematically by a system of coupled, non-linear ordinary differential equations. The variables of the system correspond to the population sizes and change over time. The transition rates describe the rate at which individuals in one compartment transit to another. Some of these rates are assumed to be constant and some are allowed to change over time describing, for example, the disease control policies that are applied in a given time period (see the Supplementary Materials for a detailed description of the set of equations, transition rates (Table S1), and compartments (Table S2) that describe the system).

The dynamics of the proposed SEAIR-severe/mild model are given as follows:

$$\frac{dS\left(t\right)}{dt}=-\alpha_{a}\left(t\right)\left(S\left(t\right)+V\left(t\right)\right)A\left(t\right)-\alpha_{i}\left(t\right)\left(S\left(t\right)+V\left(t\right)\right)I\left(t\right)-v_{e}\alpha_{a}\left(t\right)\left(S\left(t\right)+V\left(t\right)\right)A_{v}\left(t\right)+\gamma R\left(t\right)+\gamma_{v}F\left(t\right)-v_{in}\left(t\right)S\left(t\right)$$

$$\frac{dE\left(t\right)}{dt}=\alpha_{a}\left(t\right)\left(S\left(t\right)+V\left(t\right)\right)A\left(t\right)+\alpha_{i}\left(t\right)\left(S\left(t\right)+V\left(t\right)\right)I\left(t\right)+v_{e}\alpha_{a}\left(t\right)\left(S\left(t\right)+V\left(t\right)\right)A_{v}\left(t\right)-{t_{latent}^{-1}}_{}E\left(t\right)$$

$$\frac{dA\left(t\right)}{dt}={t_{latent}^{-1}}_{}E\left(t\right)-\kappa \left(t\right)A\left(t\right)-\rho A\left(t\right)$$

$$\frac{dI\left(t\right)}{dt}=\kappa \left(t\right)A\left(t\right)-\beta \;I\left(t\right)-h_{in}\left(t\right)I\left(t\right)+\left(\sigma -1\right)t_{latent}^{-1}E_{v}\left(t\right)$$

$$\frac{dR\left(t\right)}{dt}=\beta \;I\left(t\right)-\gamma R\left(t\right)+h_{out}\left(t\right)H\left(t\right)+\rho A\left(t\right)$$

$$\frac{dP\left(t\right)}{dt}=\mu \left(t\right)H\left(t\right)$$

$$\frac{dV\left(t\right)}{dt}=v_{in}\left(t\right)S\left(t\right)-v_{out}V\left(t\right)$$

$$\frac{dF\left(t\right)}{dt}=v_{out}V\left(t\right)-v_{e}\alpha_{a}\left(t\right)F\left(t\right)A\left(t\right)-v_{e}^{2}\alpha_{a}\left(t\right)F\left(t\right)A_{v}\left(t\right)-v_{e}\alpha_{i}\left(t\right)F\left(t\right)I\left(t\right)+\rho_{v}A_{v}\left(t\right)-\gamma_{v}F\left(t\right)$$

$$\frac{dH\left(t\right)}{dt}=h_{in}\left(t\right)I\left(t\right)-h_{out}\left(t\right)H\left(t\right)-\mu \left(t\right)H\left(t\right)$$

$$\frac{dE_{v}\left(t\right)}{dt}=v_{e}\alpha_{a}\left(t\right)F\left(t\right)A\left(t\right)+v_{e}^{2}\alpha_{a}\left(t\right)F\left(t\right)A_{v}\left(t\right)+v_{e}\alpha_{i}\left(t\right)F\left(t\right)I\left(t\right)-t_{latent}^{-1}E_{v}\left(t\right)$$

$$\frac{dA_{v}\left(t\right)}{dt}=\sigma t_{latent}^{-1}E_{v}\left(t\right)-\rho_{f}A_{v}\left(t\right)$$

### 2.2. Data

The model is demonstrated for Greece. The modeling parameters and outputs are constrained and optimized by the publicly available daily data provided by NOPY (https://eody.gov.gr/en/covid-19/ accessed on 15 June 2022). These data include: (i) the daily infected people, (ii) the daily number of people admitted to and (iii) discharged from the hospital, (iv) the daily number of deaths, as well as (v) the daily number of vaccinations. The total population, N, of Greece is taken from the demographic data of 2019 and is assumed to be constant throughout the simulations. Due to the fact that the model inputs are noisy measurements, we smooth the data before optimization.

We performed various averaging methods including the widely used moving average where the unweighted mean of the previous m data-points is performed and the earth moving average, which is inspired by the earth mover’s distance [35], viewing the sequence to be smoothed as a mass distribution and allowing amounts of mass to move to neighboring places in the sequence. In contrast to the earth mover’s distance, moving mass within a certain (predefined) range r is not penalized, while moving outside this predefined range is not allowed. Specifically, given a sequence x of *n* values $x_{i},i=0,\ldots ,n-1$, the smoothed sequence x is the result of an optimization procedure. The objective function of this procedure is the mean square error (MSE): $\sum_{i=0}^{n-1}{\left(x_{i}-{\underline{x}}_{i}\right)}^{2}+\lambda (\sum_{i=0}^{n-r}{\left(S({x)}_{i}-S{\left(\underline{x}\right)}_{i}\right)}^{2})$ where $S({x)}_{i}$ denotes the sum of r values of the sequence: $S({x)}_{i}$ = $\sum_{j=i}^{i+r-1}x_{j}$ and λ is a balancing factor experimentally determined (λ = 200). In practice, this objective function permits free movement of values within the range r (second term of the objective) while keeping the smoothed values ${\underline{x}}_{i}$ close to the input ones (first term). The differences between the smoothing methods are more apparent when the fluctuations in the data are steeper.

### 2.3. Model Scenarios

The baseline scenario assumes a relatively highly effective vaccine with 90% protection against serious illness, a low probability of infection or transmission upon vaccination (or infection), and an indefinite immunity period against severe illness after vaccination (or infection). Thus, for the baseline scenario, the booster shots are not considered. Building upon the baseline scenario, we predict the impact of several interesting scenarios on daily incidents, hospitalizations, and deaths, focusing on critical time-intervention points.

Specifically, we firstly explore the vaccination rate. It is important to note that the rate at which individuals become vaccinated reflects the people’s response to the vaccine campaign and comprises an unstable factor that may significantly deviate from the original vaccination plan. For that reason, at critical-intervention-time points, we varied the vaccination rate of the population accordingly to explore its impact on the spreading dynamics and disease progression informing policy-making. Secondly, vaccines can alter the course of infection [26] by providing protection against (i) infection, (ii) symptoms, (iii) severe illness, and (iv) onward transmission arising from the infected vaccinated individuals. We give particular emphasis to vaccine effectiveness to infection and onward transmission with the aim of underlining their role in disease progression. The efficacy of a vaccine is established through clinical trials conducted in controlled conditions, whereas its effectiveness is evaluated when it is administered to real populations. Thus, although clinical trials involve a diverse group of participants comprising individuals of various ages, genders, races, and medical conditions, the controlled setting may not entirely reflect the overall population. Vaccine protection against symptoms is the easiest to measure, although the definition of symptoms is often unclear or not well defined. Vaccine protection from developing severe symptoms that often require hospitalization and/or lead to death has also been well studied in clinical trials and real conditions [36,37]. On the other hand, vaccine effectiveness against infection and onward transmission is much more difficult to determine and remains a vague parameter [10,11,12,38]. Of note, vaccine efficacy and effectiveness may be considerably affected by the virus evolution. Thirdly, we investigate the loss of immunity. Several studies have shown that immunity is significantly compromised after 6 to 8 months from infection or vaccination [13,14,15].

Lastly, the final formulation of the model accounts for both the immunity loss and the booster shots, aiming to fit the complex epidemiological data throughout the disease progression. Critical evaluation regarding the different policies, the intervention measures, and the vaccination program across the various stages of the pandemic, along with the virus evolution, is demonstrated through the modeling parameters.

## 3. Results

After the initial outbreak of the pandemic, which is the phase before vaccinations (first wave), another four relatively distinct phases of the COVID-19 pandemic in Greece can be identified up to June 2022; the vaccination phase under the dominance of the wild-type strain (second wave), the vaccination phase in the rise of the delta variant (third and fourth wave), and the vaccination phase including the booster shots under the rise and dominance of the omicron variant (fifth wave). In Greece [39], both the first and the second waves were under highly strict regulation measures (i.e., social distancing measures, prevention of mobility, school closures). In the third and fourth waves, relatively looser regulation measures were present, whereas stricter regulation measures were progressively applied to these and the subsequent wave, particularly to unvaccinated individuals mainly regarding their frequent testing. Since the beginning of June 2022, Greece has lifted the COVID-19 measures. Masks are only required in hospitals, pharmacies, and health centers.

We set 1 February 2021 as the start date of our model, which corresponds approximately to the onset of the second wave in Greece and the date where data are consistently and publicly reported for Greece. The third wave becomes apparent in Greece in the middle of July 2021. The fourth wave peaks in the middle of October 2021 and the recently observed fifth wave peaks in January 2022. Data up to 15 June 2022 were used.

Notably, the model does not distinguish the incidents between vaccinated (Iv) and unvaccinated individuals (I) as these data were unavailable for most of the period under study. Thus, the transition of the vaccinated individuals from asymptomatic to confirmed cases was set equal to zero. Moreover, the model does not presume a change in the hospitalization rate due to vaccinations. This has the benefit of allow the fitting parameters to adapt according to the data and thus roughly estimate the vaccine effectiveness in the population.

### 3.1. The Invaluable Win of Vaccinations

**Figure 3:**
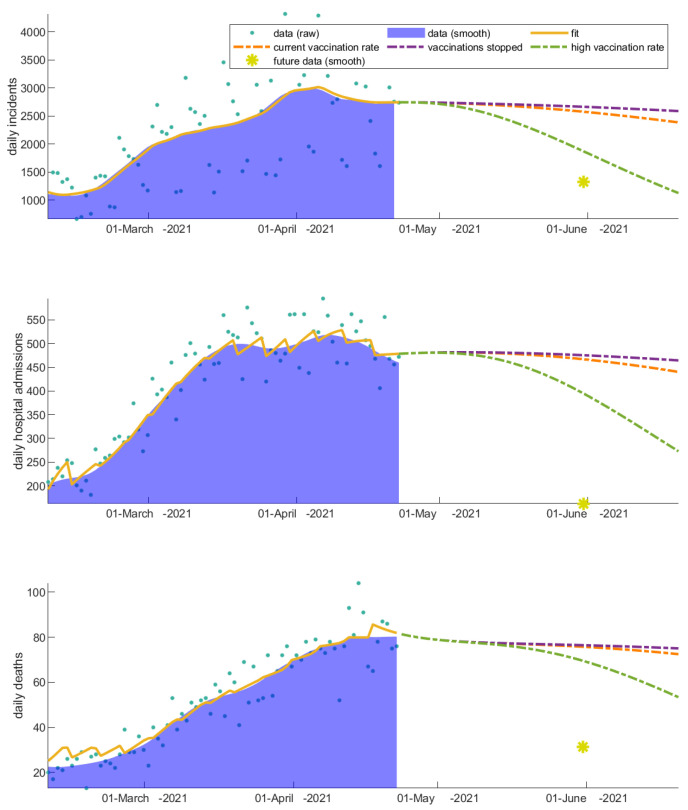
Impact of altering the vaccination rate after 23 April 2021 (decline of the second wave in Greece) on disease dynamics. The daily (a) incidents, (b) hospital admissions, and (c) deaths are highly reduced when the vaccination rate is increased. A three-month prediction is depicted. An indicative data point from the future (1 June 2021) is shown with a yellow asterisk for comparison.

**Figure 4:**
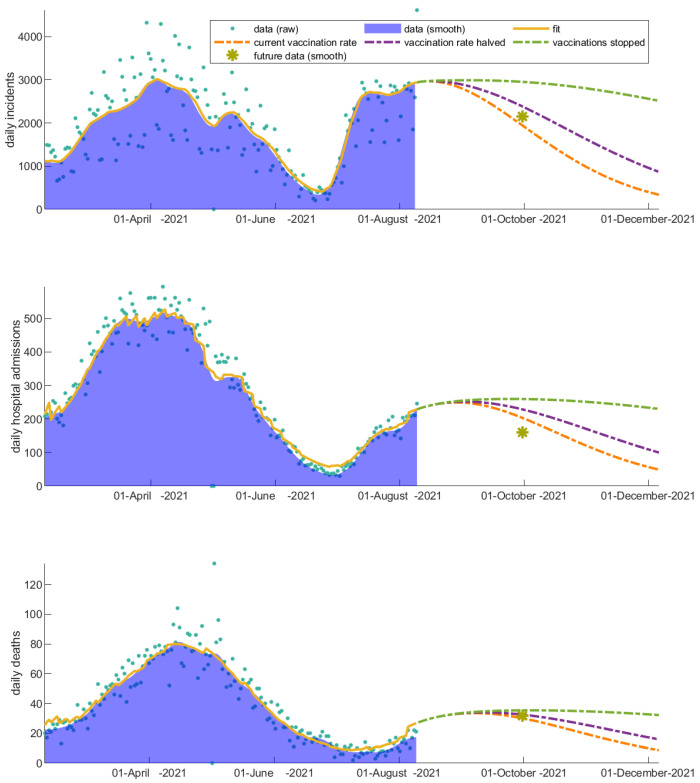
Impact of altering the vaccination rate after 10 August 2021 (rise of the third wave in Greece) on disease dynamics. The daily (a) incidents, (b) hospital admissions, and (c) deaths are affected indicating the importance of keeping up the high vaccination pace. An indicative data point from the future (1 October 2021) is shown with a yellow asterisk for comparison.

We explore the impact of vaccinations by altering the vaccination rates (i) during the decline of the second wave and (ii) during the rise of the third wave in Greece, in order to understand how the rate of community response to vaccinations regulates the rise and fall of the pandemic waves and affects hospitalizations and deaths. We first apply these scenarios choosing the date of changing the vaccination rates to be 23 April 2021. Ignoring the onset of the following wave, the high vaccination rate, which is an approximation of the true rise of the vaccination rate, better describes the decline of the current wave under study—indicating how important the response of the people was to the vaccination programs at that particular point in time (Figure 3). However, as long as vaccinated individuals become infected, the hospitalization rate decreases significantly; this explains the discrepancy we actually observe in the projected hospitalizations and deaths in the future time point (Figure 3). The observed discrepancy is true evidence of the win of vaccinations. We next apply the vaccination scenarios after 10 August 2021, which corresponds to the onset of the third wave in Greece (Figure 4). As this scenario is later in time, the effect of vaccinations on mortality and hospital admissions has been already absorbed in the related fitting parameters of the model. Thus, the future projection is closer to the real data for that particular case.

The importance of sustaining the vaccination rate at a high level has a drastic impact on hospitalizations and deaths, even in the decline of the epidemic wave. The predicted increase on cumulative incidents, hospitalizations, and deaths over the next 90 days from 10 August 2021 in the hypothetical scenario where vaccinations suddenly stop is depicted in Table 1.

### 3.2. Vaccinated Individuals Play a Catalytic Role on COVID-19 Progression

We next investigate how different values of vaccine protection to infection and onward transmission affect the future projection of incidents, hospitalizations, and deaths.

**Figure 5:**
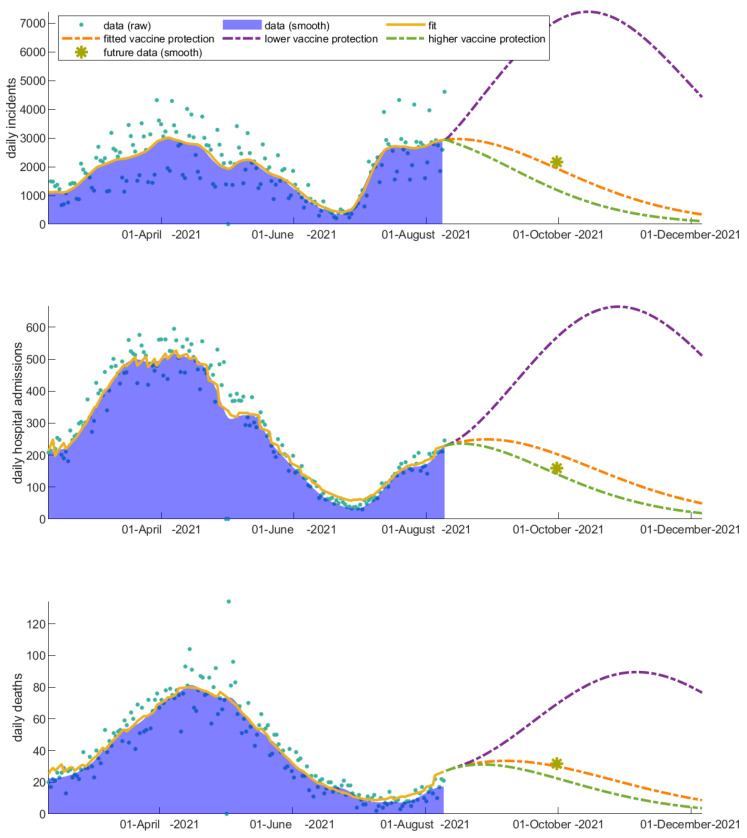
Impact of vaccine effectiveness against infection and transmission on driving the disease dynamics after 10 August 2021. The daily (a) incidents, (b) hospital admissions, and (c) deaths are considerably affected indicating the importance of self-awareness in vaccinated individuals. An indicative data point from the future (1 October 2021) is shown with a yellow asterisk for comparison.

As shown in Figure 5, an increase in the infection/transmission parameter of the vaccinated individuals drastically increases the daily incidents and prolongs the wave, sharply increasing the hospitalizations and the people that eventually succumb to the virus. An indicative data point set on 1 October 2021 is also shown in Figure 5 for comparison. Considering that the parameters describing the disease progression only slightly vary, a vaccine protection to infection and onward transmission closer to the baseline value best predicts the future dynamics for that particular time period.

Increasing the probability of the onward transmission of the virus from vaccinated individuals had one of the largest impacts, counting for 357,227, 26,357 and 2960 more incidents, hospitalizations, and deaths, respectively, over a period of three months relative to the baseline scenario (Table 1), questioning the measures selectively applied to vaccinated individuals for that particular time period. Fortunately, despite the increased transmissibility of the emergent strains, the severity of the virus was significantly compromised.

### 3.3. Immunity Loss Feeds Back the Severity of the COVID-19 Waves

If immunity is compromised and it is not supported by timely booster shots, we predict an increase in the total incidents, hospitalizations, and deaths equal to 135,823, 8516 and 2909, respectively, within a period of three months (Table 1).

**Figure 6:**
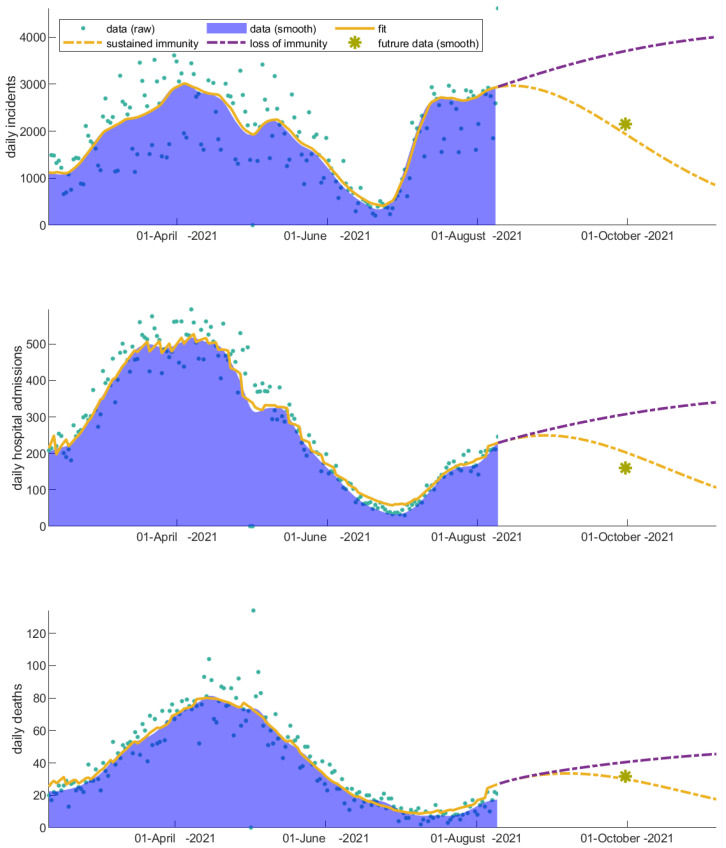
Impact of immunity loss on the disease dynamics after 10 August 2021. The daily (a) incidents, (b) hospital admissions, and (c) deaths are shown. An indicative data point from the future (1 October 2021) is shown with a yellow asterisk for comparison.

As expected, immunity loss highly increases the susceptible population feeding back into the spreading dynamics (Figure 6). An indicative future data point set on 1 October 2021 is also shown in Figure 6. Note that the booster shots were initiated after 1 October 2021 in Greece. We can thus argue that for that particular time period and for the dominant strain at present, immunity is still in place in the population. However, as we shall discuss later, in late October 2021, the delta variant prevailed in Greece, giving rise to the fourth wave and—together with the rise of omicron—changing the importance of booster shots.

Concluding over all the hypothetical scenarios presented (summarized in Table 1), on 10 August 2021, it was critical to increase the vaccination coverage in the population and initiate the booster shot program in order for people to become protected from severe illness, even when infections after vaccination are highly probable.

### 3.4. Evaluation of Vaccinations throughout COVID-19 Evolution

**Figure 7:**
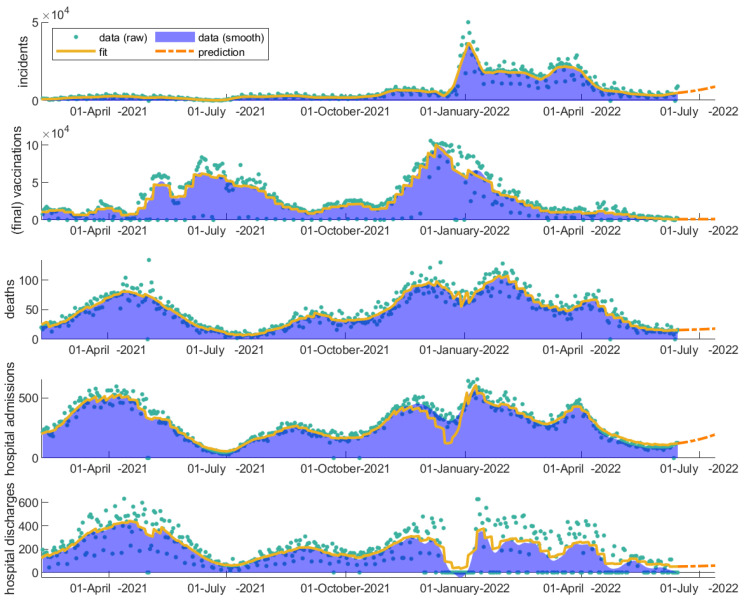
Real and fitted data for Greece from daily incidents, vaccinations, deaths, admission to and discharges from the hospitals for the fitting time period 1 February 2021–15 June 2022. The real data are shown with blue dots. The model fitting is shown with a continuous line and the future two-week predictions are shown with a dashed line.

In order to describe and understand all the waves since 1 February 2021 up to 15 June 2022, the model additionally accommodated the booster shot program and the immunity loss of vaccinated and recovered unvaccinated people after a period of 6 months. The modeling fitted data corresponding to the daily incidents, vaccinations, deaths, admission to and discharges from the hospitals for the time period 1 February 2021–15 June 2022 are shown in Figure 7, along with the real data.

**Figure 8:**
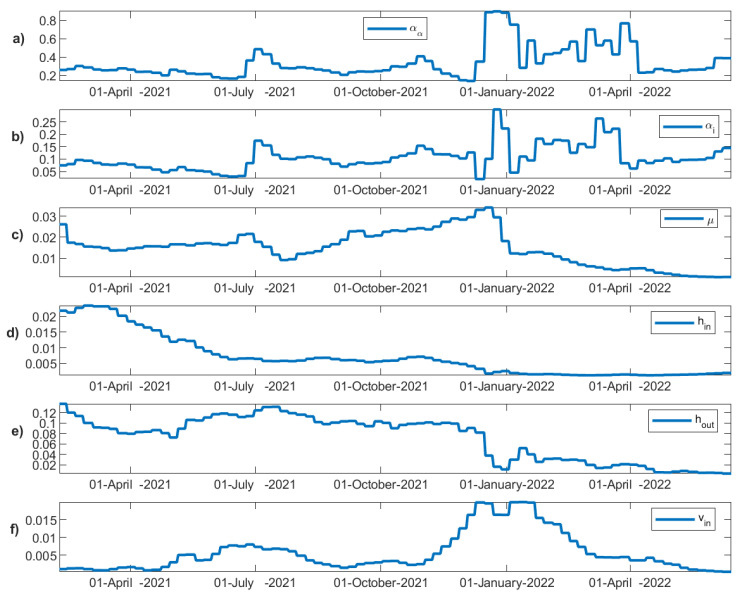
Fitted parameters over time for the fitting time period 1 February 2021–15 June 2022. (**a**) Transmission rate of asymptomatic individuals (a_a_), (**b**) transmission rate of confirmed cases (a_i_), (**c**) mortality rate (μ), (**d**) admission rate to hospitals (h_in_), (**e**) discharge rate from hospital (h_out_), and (**f**) vaccination rate over time (v_in_).

The regulation measures, the daily incidents, the vaccinations, and the dominant strains vary considerably throughout the progression history of the disease. These changes are reflected in the modeling parameters. The fitted values of each of the time-varying transition parameters are depicted in Figure 8. The transmission rates of asymptomatic and confirmed incidents vary similarly through time, although at different ranges, capturing the timeline of the different waves of the SARS-CoV-2 virus observed in Greece (Figure 8a,b). Initially, a decrease over time is observed with small fluctuations reflecting the various yet strict regulation measures taken in Greece during the second wave. A rapid outburst in the middle of July 2021 then follows, dictating the beginning of the third wave. Due to the relaxed regulation measures and the onset of the delta variant in Greece, the transmission rates during that period remain considerably high. In late October 2021, the rise of the fourth wave once again under the dominance of the delta variant can also be observed. Finally, at the end of December 2021, a very steep increase in the transmission rates (1.5 times higher than the transmission rate of the delta variant in the middle of July) becomes apparent, confirming the dominance of the highly transmissible omicron variant in the population. The wave of the omicron strain and its variants that appeared thereafter lasted for more than four months in Greece and its dominance remained continuous with subsequent waves.

**Figure 9:**
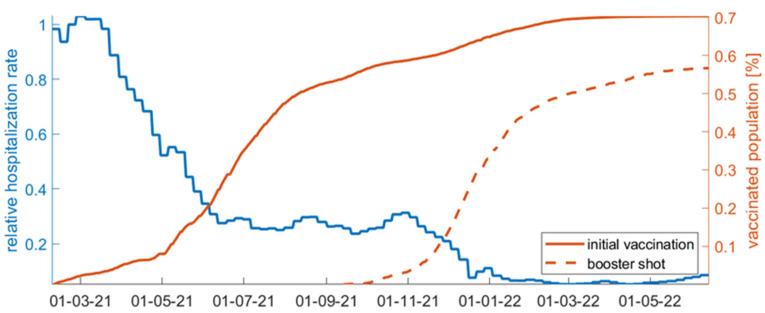
Relative hospitalization rate (calculated by dividing the hospitalization rate after vaccinations by the hospitalization rate before vaccinations) depicted with the blue line against the percentage of vaccinated individuals in the population (depicted with the orange line) for the time period 1 February 2021–15 June 2022. The percentage of individuals who have received a booster shot is also shown (dashed orange line).

Admissions to hospitals are initially high, decreasing over time (Figure 8d). After July 2021, they remain at low rates even when the daily incidents escalate and while the regulation measures remain relatively looser, supporting the significance of vaccinations towards avoiding severe illness and health system overload. The discharge rate from the hospital remains relatively constant, fluctuating around 0.1d-1 until December 2021 (Figure 8e). The mortality rate (Figure 8c), reflecting the rate at which hospitalized patients eventually succumb to the disease, fluctuates between 0.01 and 0.03d-1. The mortality rate is highly dependent on virus severity, the level of immunity, advanced age, the presence of co-morbidities, and genetics, among other reasons. An alarming increase in death rate from August 2021 to December 2021 is observed despite the low rate of admissions to the hospital, possibly indicating a cumulative overload in the intensive care units or loss of immunity or both. Interestingly, after December 2021, the mortality rate decreases substantially, which coincides with the steep increase in the booster vaccinations. After January 2022, the dominance of the omicron variant also becomes apparent in the mortality rate, keeping it at significantly low levels. Regarding vaccinations (Figure 8f), since 1 February 2021 and for the first two months, the vaccination rate in Greece remains at relatively low rates. Then, it increases substantially up to the middle of July 2021. A rate decline is then observed with the percentage of fully vaccinated people in Greece having reached 60% of the population up to October 2021 and 70% up to June 2022. At the end of September 2021, the booster shot program was initiated (Figure 9).

Up to September 2021, the available data for Greece do not separately report the number of vaccinated and unvaccinated infected individuals. It is expected that a proportion of the reported daily incidents correspond to vaccinated infected individuals. We hypothesize that this fact must be reflected in the admission rate to the hospitals. In particular, assuming that vaccinations protect infected individuals from hospitalization with a specific effectiveness, then, in the mean field approximation and with no virus evolution, the hospitalization rate should actually reflect the ratio between the protected and unprotected, high-risk infected individuals. As can be seen in the relative hospitalization rate (hospitalization rate after vaccinations divided by the hospitalization rate before vaccinations) in Figure 9, there were initially many more unprotected individuals that gradually became protected, resulting in a significant decline of the hospitalization rate over time. We can observe that the hospitalization rate has reached a plateau since June 2021, decreasing no further until October 2021 despite the increase in vaccinations; this observation shows that a relatively constant percentage of high-risk individuals remain unprotected, either because they do not get vaccinated, their immunity has critically declined, the vaccination protection during the rise of new strains has been compromised, due to serious comorbidities, or because of any combination of the aforementioned hypotheses. However, after November 2021, a significant decline in the admission rate to hospitals is observed, which coincides with the increased number of booster shots in the population.

**Figure 10:**
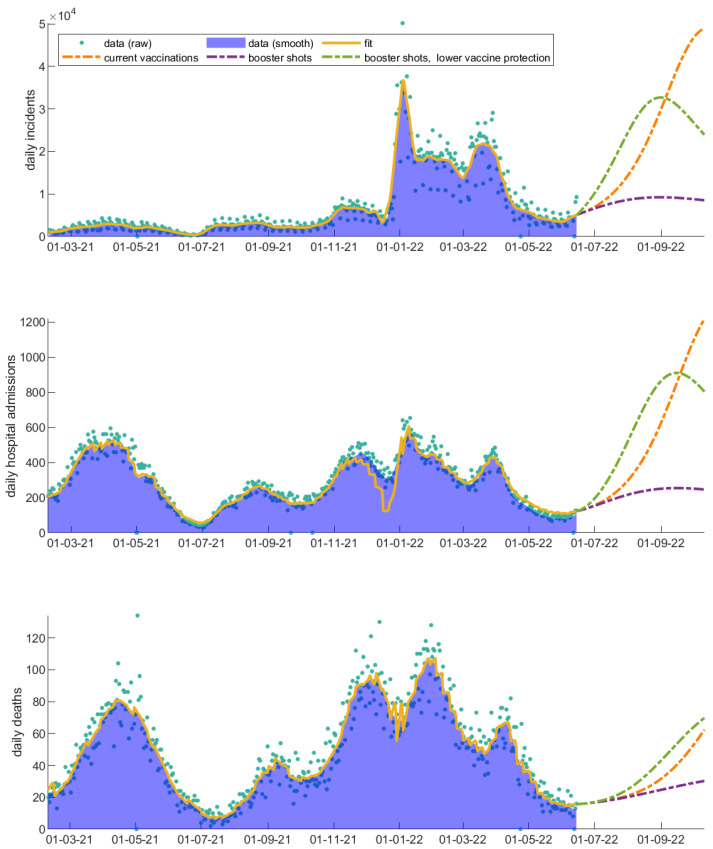
The impact of an effective booster shot is shown under the dominance of omicron sub-variants. The fitting time period is 1 February 2021–15 June 2022. The daily (a) incidents, (b) hospital admissions, and (c) deaths are shown.

Since May 2022, most restrictions have been lifted in Europe. Shortly after the relaxation of measures, the omicron strain revived through its sub-variants (BA.4 and BA.5), hitting again with a new wave which also became apparent in Greece. Even though the mortality rate and hospitalization rates have significantly decreased under the dominance of the omicron strain, a rapid outburst of incidents can increase the hospitalizations and deaths. As the model shows, an effective booster shot probably more targeted to the omicron sub-variants might help the more sensitive individuals. Figure 10 shows the projection of the newly coming wave and its impact on incidents, hospitalizations, and deaths under the current conditions (Figure 10, orange dotted line). An effective booster shot can significantly decrease the coming challenge (Figure 10, purple dotted line), even when the vaccine protection to infection and onward transmission is compromised (Figure 10, green dotted line).

## 4. Discussion

A compartmental SEIR-type model is utilized in this work to monitor and study the spread dynamics of COVID-19 throughout its complex evolution, in order to guide policy-making at critical time-intervention points and assess vaccine effectiveness as the virus evolves. The vaccination rate and immunity loss, along with the vaccine protection and virus transmissibility rates, which are both subject to virus evolution, are considered together. Based on the modeling predictions, regulation measures could be decided to relieve the healthcare system overload and protect the population when necessary. The model is demonstrated for Greece. The modeling parameters and outputs are constrained and optimized by the publicly available daily data for the time period between 1 February 2021 and 15 June 2022, which covers the period before and during the vaccinations and where the virus has evolved to variants of significant concern.

An important decrease in the admission rate to the hospitals has been observed as the pandemic progresses, highlighting the significant role that vaccinations play in preventing severe illness. We have further demonstrated that vaccinations drastically reduce spread dynamics and hospital admissions, even when vaccinated individuals are allowed to become infected and transmit the virus contributing to virus circulation. Our results show that vaccinated people comprise critical players in the spreading dynamics, thus self-awareness and frequent testing must apply to the entire population. Considering that immunity declines over time for both vaccinated and recovered individuals, the results of this study underlie the importance of booster shots. In support of this, when fitting the model to the real data, an alarming period with increased deaths just before the initiation of the booster shot program has been revealed under the dominance of the delta variant in Greece.

Overall, there exist several mechanisms that can influence the spreading dynamics and alter the ratio of individuals who are more likely to experience severe symptoms (severe path) compared with those who experience milder symptoms (milder path). *S* represents the group of susceptible individuals who, if infected by the virus, will most likely experience severe symptoms (severe path). Changes in vaccination rates directly impact the *S* compartment. When the transmissibility parameter of affected vaccinated individuals (*v_e_*) is changed (as shown in Figure 5), the disease’s spread dynamics are activated but the S compartment is not directly affected. Instead, the mild and severe paths are activated by increasing the number of exposed individuals (*E* and *E_v_* compartments). The final formulation (Figure 7) takes into account immunity loss, which leads to individuals moving from the *F* to the *S* compartment but also accounts for the booster shots, which lead to individuals moving back from the *S* compartment to the *F* compartment. Certainly, with the added consideration of virus evolution, the objective is to shift all members of a population towards experiencing milder symptoms (milder path).

Our findings are also subject to several limitations. The proposed model assumed the homogeneous mixing of individuals in the compartments, ignoring individual-level variations or age-structured compartments, spatial effects, and social networks that may drive the spreading dynamics to different regimes and may allow an even deeper understanding of how individuals under specific circumstances affect and are affected by the pandemic. Greece is a country characterized by an unbalanced spatial distribution of its population, with an over-concentrated population in the major urban centers. On top of that, the vast arrival of non-residents at tourist destinations that is spread out unevenly over the year also establishes an unbalanced seasonal-dependent spatial distribution of the population. The model design and fitting capability are also constrained to the data available. Thus, although the data allow more realistic scenarios to be explored, they are limited. More specifically, hidden states including the asymptomatic population, as well as the number of vaccinated/unvaccinated incidents and hospitalized patients that affect both the model parameterization and the accuracy of the predictions, remain publicly unknown. With the data currently available, the predicted effect of vaccinations on the population remains strongly coupled with the virus evolution. As more data are collected and become publicly available, more precise models will be built.

## 5. Conclusions

Vaccines are one of the most effective public health measures for controlling the spread of pandemics, including SARS-CoV-2. By incorporating the realistic vaccination program implemented in Greece into the model, this study provides a more accurate and comprehensive understanding of how policy decisions affect the spread of COVID-19. Overall, our findings highlight the importance of booster shots and the continuation of protective measures, even among the vaccinated population, in order to control the spread of COVID-19.

With the emergence of new COVID-19 variants and the inadequacy of vaccine protection in reducing transmission, it is imperative to closely monitor the intricate evolution of both the virus and the vaccines to enable a proactive response in the future. Given the current scenario, we must enhance our vaccination-based strategy while also prioritizing the development of effective therapeutics. Moreover, a comprehensive understanding of the complex pathophysiology and risk factors associated with the disease is necessary to combat this pandemic.

## Figures and Tables

**Table 1:** Impact of each scenario on the cumulative number of incidents, hospitalizations, and deaths (over the next 90 days after 10 August 2022) relevant to the baseline scenario.

Scenarios	Increase in Number of Incidents	Increase in Number of Hospitalizations	Increase in Number of Deaths
Vaccinations stop	77,326	4792	501
Infection after vaccination becomes twice as probable	357,227	26,357	2960
Vaccine protection to severe illness is compromised to 10%	31,101	8966	1068
Immunity is lost in 6 months	135,823	8516	909

## Data Availability

The data analysis presented in this study is available upon reasonable request from the corresponding author.

## Supplementary Materials

The following are available online at https://www.mdpi.com/article/10.3390/vaccines11040722/s1, Table S1: Model parameters, Table S2: Model compartments.
