# Supplementary material for: Mathematical Modeling Evaluates How Vaccinations Affected the Course of COVID-19 Disease Progression
Source: Vaccines (Basel). 2023 Mar 24;11(4):722. doi: 10.3390/vaccines11040722 (PMC10142609; doi:10.3390/vaccines11040722)
Supplement: Supplementary file 1 [file vaccines-11-00722-s001.zip › vaccines-2284504-supplementary.pdf]

Supplementary Methods

Adapted and modified SEIR model

COVID-19 spread dynamics are described through adaptations of a SEIR compartmental model(1, 2). In this broadly used framework, the whole population is divided into homogeneous (mean-field approximation) groups, usually called compartments. Individuals transit between compartments, but can only be in one compartment at a given time point. In SEIR models, the whole population N is divided into: i) a susceptible population (S), ii) exposed individuals (E), which have been infected but are not yet infectious (considering a latency period), iii) infected people (I), and iv) recovered or removed people (R), which correspond to people that have either recovered or perish from the disease.

In our work, the whole population is split into two main SEIR-type branches in which the first describes a potentially more severe progression and the other branch describes a milder progression of the disease. We consider S as a compartment that accommodates pure susceptible individuals; these are individuals susceptible to both branches. Depending on the vaccination rate, the susceptible population progressively becomes vaccinated and in general follows the milder progression path. These individuals can be also infected yet, with milder symptoms, and can also spread the disease. Assuming a certain vaccine inefficacy to severe illness, some of these individuals may follow the more severe branch.

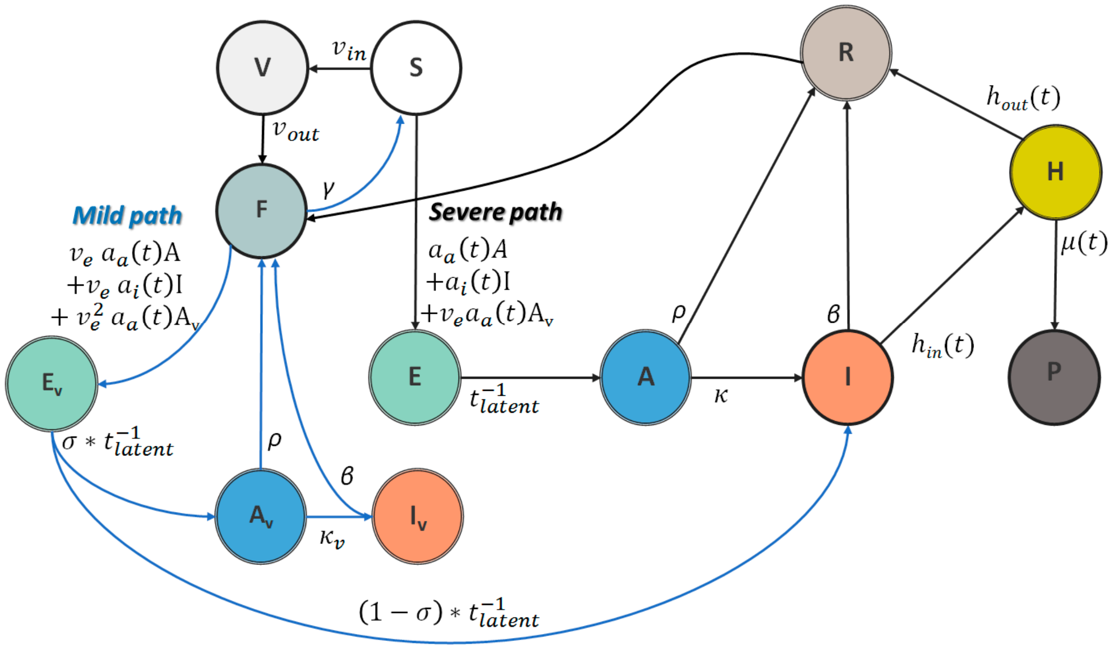

Figure S1 *Schematic overview of the proposed model (SEAIR-severe/mild).*

In more detail, we assume that the infected population is composed of those individuals that have been confirmed as COVID-19 patients (I) and those that are asymptomatic (A) and remain undetected from the system. Furthermore, we distinguish between the recovered individuals (R) and those that perish from the disease (P). In addition, we assume that confirmed patients can either recover at home (mild incidents) or be hospitalized (severe incidents). Furthermore, we take into account the vaccinated individuals including other two additional compartments; the vaccinated (V) and the immune (F). Specifically, we assume that the vaccinated individuals are those who have completed the initial vaccination protocol (a second dose of a two-dose vaccine like the Pfizer/BioNTech and Moderna vaccine, or a first dose of a one-dose vaccine like the Johnson & Johnson vaccine). Vaccinated people are considered immune after a period of time, where the human body reaches a threshold of protection. In other words, the immune population is the vaccinated population after a certain period of time. Note that before that time the vaccinated population, V,

is considered susceptible. The booster shot (a third dose of Pfizer/BioNTech vaccine, or a second dose of Johnson & Johnson vaccine) is also accommodated in the model. In that case, susceptible individuals are directly transferred to the immune population with a rate equal to the vaccination rate of the third dose. The vaccinated (and recovered) individuals are susceptible to infection, yet progress to the mild path instead, as exposed (E_v) and asymptomatic (A_v). The vaccinated confirmed cases (I_v) are not considered in the current version due to lack of related data. Furthermore, recovered and vaccinated individuals can become susceptible to severe illness again due to loss of immunity(3-5). A schematic illustration of the proposed model is shown in **Figure S1**.

In terms of the mathematical basis, the model is described by a system of coupled, non-linear ordinary differential equations. The variables of the system correspond to the population sizes and change over time. The transition rates describe the rate at which individuals in one compartment transit to another. Some of these rates are assumed constant and some are allowed to change over time describing for example the disease control policies that are applied at a given time period. The time-varying parameters include the a_a and a_i, which reflect respectively the probability of the asymptomatic/unconfirmed (A) and the confirmed infected (I) to infect the susceptible population; parameters that are highly affected by social distancing measures, shelter-in-place strategies, and the strain variant. The transition rate κ from asymptomatic/unconfirmed to (confirmed) infected population is assumed constant and reflects the frequency of testing. The asymptomatic population recovers with rate ρ. The infected population can either recover directly with rate $\beta = \rho$ or be hospitalized with rate h_{in}. The hospitalized population either recovers with rate h_{out} or succumbs from the virus with mortality rate μ. The parameters h_{in},$h_{out}$and μ are considered time-varying parameters to enable them to express the potential role of vaccinations and virus evolution on hospitalizations and mortality. The susceptible population becomes vaccinated (V) at rate $v_{in}$and is rendered immune (F) at rate v_{out}. The vaccination rate v_{in} is also a time-varying parameter. The recovered population can become susceptible again (loss of immunity) with a constant rate γ. We also consider the case where the immune population (F and R) can become infected (Ev) from either asymptomatic (A), confirmed patients (I) or other infectious immune people (Av) with vaccine effectiveness against infection reduced by a factor v_e, respectively for each transmission rate. The infected immune population recovers with rate ρ and can also spread the disease contaminating the susceptible population (S) with effectiveness against transmission reduced by a factor v_e relative to the unvaccinated population. Based mainly on the vaccine effectiveness (σ) against serious illness, the infected immune people may transit to the more severe path. Waning immunity over time has been also observed in vaccinated individuals. Thus, vaccinated people become susceptible again with rate γ. Part of the infected vaccinated individuals may also become detected incidents (Iv), although we do not directly account for this scenario due to current lack of related data. All the parameters and variables of the proposed models are depicted in **Table S1** and **Table S2**, respectively.

The dynamics of the proposed SEAIR-severe/mild model are given as follows:

$$\frac{dS(t)}{dt} = -\alpha_a(t)\big(S(t) + V(t)\big)A(t) - \alpha_i(t)\big(S(t) + V(t)\big)I(t) - v_e\, \alpha_a(t)\big(S(t) + V(t)\big)A_v(t) + \gamma R(t) + \gamma_v F(t) - v_{in}(t)S(t)$$

$$\frac{dE(t)}{dt} = \alpha_a(t)\big(S(t) + V(t)\big)A(t) + \alpha_i(t)\big(S(t) + V(t)\big)I(t) + v_e\, \alpha_a(t)\big(S(t) + V(t)\big)A_v(t) - t_{latent}^{-1}\, E(t)$$

$$\frac{dA(t)}{dt} = t_{latent}^{-1}\, E(t) - \kappa(t)A(t) - \rho\, A(t)$$

$$\frac{dI(t)}{dt} = \kappa(t)A(t) - \beta\, I(t) - h_{in}(t)\, I(t) + (\sigma - 1)t_{latent}^{-1}\, E_v(t)$$

$$\frac{dR(t)}{dt} = \beta\, I(t) - \gamma R(t) + h_{out}(t)H(t) + \rho\, A(t)$$

$$\frac{dP(t)}{dt} = \mu(t)H(t)$$

$$\frac{dV(t)}{dt} = v_{in}(t)\, S(t) - v_{out}\, V(t)$$

$$\frac{dF(t)}{dt} = v_{out}\, V(t) - v_e\, \alpha_a(t)F(t)A(t) - v_e^2\, \alpha_a(t)F(t)A_v(t) - v_e\alpha_i(t)F(t)I(t) + \rho_v A_v(t) - \gamma_v F(t)$$

$$\frac{dH(t)}{dt} = h_{in}(t)I(t) - h_{out}(t)H(t) - \mu(t)H(t)$$

$$\frac{dE_v(t)}{dt} = v_e\, \alpha_a(t)F(t)A(t) + v_e^2\, \alpha_a(t)F(t)A_v(t) + v_e\alpha_i(t)F(t)I(t) - t_{latent}^{-1}E_v(t)$$

$$\frac{dA_v(t)}{dt} = \sigma\, t_{latent}^{-1}E_v(t) - \rho_f\, A_v(t)$$

Table S1 *Model parameters*

Name	Range	Description
$a_a(t)$	[0.001, 0.9] [days^{-1}]	probability of infection by asymptomatic/unconfirmed incidents [**estimated time-varying parameter**]
$a_i(t)$	[0.01, 0.3] [days^{-1}]	probability of infection by confirmed incidents [**estimated time-varying parameter**]
κ	1/2 [days^{-1}]	transition rate from asymptomatic to confirmed incidents: testing rate [**bibliographically given constant parameter**]
ρ	1/6 [months^{-1}]	recovery rate of asymptomatic and vaccinated individuals [**bibliographically given constant parameter**]
β	1/6 [months^{-1}]	recovery rate of confirmed incidents [**bibliographically given constant parameter**]
$h_{in}(t)$	[0.001, 0.5] [days^{-1}]	admission rate to hospital [**estimated time-varying parameter**]
$h_{out}(t)$	[0.001, 0.3] [days^{-1}]	discharge rate from hospital [**estimated time-varying parameter**]
$\mu(t)$	[0.0001, 0.05] [days^{-1}]	mortality rate of hospitalized [**estimated time-varying parameter**]
$v_{in}(t)$	[0.0001, 0.02] [days^{-1}]	vaccination rate of susceptible population [**estimated time-varying parameter**]
v_{out}	1/14 [days^{-1}]	latency rate for full immunity of vaccinated population **[bibliographically given constant parameter]**
γ	1/6 [months^{-1}]	immunity loss rate **[bibliographically given constant parameter]**
v_e	[0, 1]	vaccine effectiveness against infection factor **[unknown parameter]**
σ	[0, 1]	vaccine effectiveness against serious illness **[unknown parameter]**
t_{latent}	2 days	latent period of the virus after exposure **[bibliographically given constant parameter]**

Table S2 *Model compartments*

Compartment	Description
S	Susceptible population to both mild and severe branches
E	Exposed to the virus, but not yet infectious population

A	Asymptomatic and infectious population
I	Confirmed infected population
R	Recovered population (originating from A, I, or H)
P	Population succumbed to the disease
V	Vaccinated but not yet fully immune population
F	Vaccinated and fully immune population
H	Hospitalized population
E_v	Immune population exposed to the virus
A_v	Immune population, which is asymptomatic and infectious

The model variables and parameters are optimized to fit the data for a given time-period. This is commonly known as a *fitting* or *optimization process.* After the optimization process, the transition rates (parameters) are kept fixed and allow the model to make future predictions, by evolving the population of the compartments (variables) under the assumption that these parameters do not change during the time window of the predictions. This is called the *prediction* or *projection process*.

Fitting is important: i) for assessing whether the model is capable to describe the data observed, ii) for allowing understanding of the underlying mechanisms involved (i.e. impact of regulation measures, seasonal and behavioral changes, emergence of new strain variants), and iii) to realize predictions based on the history of the disease evolution to inform policy making.

Data

The model is demonstrated for Greece. The modeling parameters and outputs are constrained and optimized by the publicly available daily data provided by NOPY(6). The data include: i) the daily infected people, ii) the daily admitted to iii) and discharged from the hospital, iv) the daily number of deaths, as well as v) the daily number of vaccinations. The total population, *N*, of Greece is taken from the demographic data of 2019 and is assumed constant throughout the simulations.

Data pre-processing

Due to the fact that the model inputs are noisy measurements (i.e. drops in counts are commonly observed during the weekend as less tests and vaccinations are performed), we smooth the data before the optimization. We performed various averaging methods including the widely used moving average where the unweighted mean of the previous m data-points is performed and the earth moving average, which is inspired by the earth mover's distance(7) viewing the sequence to be smoothed as a mass distribution, and allowing amounts of mass to move to neighboring places in the sequence. In contrast to the earth mover's distance, moving mass within a certain (predefined) range r is not penalized, while moving outside this predefined range is not allowed. Specifically, given a sequence x of n values $x_i, i = 0, \dots, n-1$, the smoothed sequence $\underline{x}$ is the result of an optimization procedure. The objective function of this procedure is the Mean Square Error (MSE): $\sum_{i=0}^{n-1} \quad (x_i - \underline{x}_i)^2 + \lambda(\sum_{i=0}^{n-r} \quad (S(x)_i - S(\underline{x})_i)^2)$, where $S(x)_i$ denotes the sum of r values of the sequence: $S(x)_i = \sum_{j=i}^{i+r-1} \quad x_j$ and λ is a balancing factor experimentally determined ($\lambda = 200$). In practice, this objective function permits free movement of values within the range r (second term of the objective) while keeping the smoothed values $\underline{x}_i$ close to the input ones (first term). The differences between the smoothing methods are more apparent when the fluctuations on the data are steeper.

Variable and parameter estimation

We assume an initial state, which describes the starting point of our simulations. Hospitalized, dead, infected and vaccinated compartments are initialized based on the current related epidemiological data. The numbers of exposed, asymptomatic and infected subjects are initialized arbitrarily to be equal to 100/N, 1000/N and 1000/N, respectively. While the recovered compartment is initialized with the cumulative incidents up to the first day of the fit minus the population of compartment I. The number of dead people is initialized with the number of the cumulative deaths up to the first day of the fitting. The hospitalized compartment is initialized as the $h_{in}(0)$ multiplied by the initial value of compartment I. Considering that we begin the fitting process at a time when numbers of vaccinated people were negligible, we assume that the fully immune people from vaccinations are initially considered equal to 0. Instead, we take them into account by setting the initial number of vaccinated but not fully immune (compartment V) as the total vaccinated people at the first day of the fit. The exposed vaccinated and the asymptomatic vaccinated compartments are initially assumed to be empty. The initial population of the susceptible compartment S is the total population minus all the other compartments.

Both the fixed and the parameters to be fitted are depicted on **Table S1**. The time-varying parameters are constrained to be piecewise constants over certain intervals as in Tsay et. al.(2) to avoid overfitting. Bounds on their values are given to the parameters as shown in Table I. The set of all the parameters that are not given *a priori* and the variables that describe the system are optimized based on the available data. The commonly used least-squares regression method is used with the objective to minimize the MSE between simulations and measured values, thus best fit the data set. A weighted loss function is used to penalize differently the error terms.

Model scenarios

In the baseline scenario, we assume that σ, v_e and γ are equal to 0.9, 0.25, and 0, respectively. The baseline scenario thus assumes a relatively highly effective vaccine with 90% protection against serious illness, low probability of infection or transmission upon vaccination (or infection), and an indefinite immunity period against severe illness after vaccination (or infection). Thus, for the baseline scenario the booster shots are not considered.

Focusing on critical time-intervention points, we then explore several predictive scenarios including the impact of i) various vaccination rates, ii) the vaccine effectiveness v_e describing the protection of vaccinated individuals against infection and spread, and iii) the loss of immunity as reflected in the parameter γon daily incidents, hospitalizations and deaths.

The COVID-19 vaccination program in Greece followed similar prioritization protocols(8) with other countries in Europe. Taking into account the limited supply of vaccines, an initial prioritization was given to health care workers and medically vulnerable individuals. Age-structured prioritization with higher priority to senior citizens then followed. We fitted the model to the existing vaccination rate in Greece based on the publicly available data assuming, however, well-age-mixing in the population. It is important to note that the rate at which individuals get vaccinated reflects the people's response to the vaccine campaign and comprises an unstable factor that may significantly deviate from the original vaccination plan. For that reason, at critical-intervention-time points, we varied accordingly the vaccination rate of the population to explore its impact on the spreading dynamics and disease progression informing policy making.

Additionally, we give particular emphasis on vaccine effectiveness to infection, onward transmission, and protection against severe illness with the aim to underline their role in disease progression. Considering that the current available data for Greece did not provide information of whether the reported incidents, hospitalizations and deaths result from vaccinated or unvaccinated individuals, reported or hypothetical values of the corresponding variables and parameters can be only considered. The role of vaccine efficacy/effectiveness against severe illness has been more extensively studied in laboratory/real conditions(9, 10). On the other hand, the vaccine effectiveness against infection and onward transmission is

highly more difficult to determine and it still remains a vague parameter(11-14). Of note, vaccine efficacy and effectiveness may considerably be affected by the virus evolution. In general, we assume that vaccinated people can be infected and transmit the virus similarly to asymptomatic people. Specifically, we assume that the vaccine effectiveness against infection and transmission is reflected on the reduced factor $v_e \in [0,1]$ relative to the asymptomatic unvaccinated population. In general, v_e can be different for infection relative to transmission, but for simplicity we assume the same value for both cases. v_e closer to zero reflects lower probability of a vaccinated person to be infected and transmit the virus, whereas v_e closer to one actually assumes no difference between vaccinated and unvaccinated asymptomatic people. The vaccine effectiveness against infection and transmission might also vary over time particularly when different regulation measures are applied among vaccinated and unvaccinated people in the community and may change in the presence of new strains. We vary v_e to lower and higher values and explore its impact on daily incidents, hospitalizations and deaths. The vaccine effectiveness against severe illness (σ) may also be compromised in the emergence of a novel, highly aggressive variant of the virus.

At last, a long debate has emerged regarding the waning immunity after vaccination or infection. Recent studies have shown that immunity is significantly compromised after 6 to 8 months from infection or vaccination(3-5). Furthermore, new studies(5) have also shown that older individuals given a third booster dose of vaccine were less likely to develop severe illness than those who had not received the boosters. Therefore, the immunity loss rate γ, is also explored in this study as it may considerably revive the susceptible population with drastic consequences in virus spread. In the final formulation of the model, we assume that immunity is lost in 6 months, whereas the booster shots are also considered.

References

1. Chowell G. Fitting dynamic models to epidemic outbreaks with quantified uncertainty: A Primer for parameter uncertainty, identifiability, and forecasts. Infectious Disease Modelling. 2017;2(3):379-98. Epub 2017/12/19.
2. Tsay C, Lejarza F, Stadtherr MA, Baldea M. Modeling, state estimation, and optimal control for the US COVID-19 outbreak. Scientific reports. 2020;10(1):10711. Epub 2020/07/03.
3. Dan JM, Mateus J, Kato Y, Hastie KM, Yu ED, Faliti CE, et al. Immunological memory to SARS-CoV-2 assessed for up to 8 months after infection. Science (New York, NY. 2021;371(6529). Epub 2021/01/08.
4. Feikin DR, Higdon MM, Abu-Raddad LJ, Andrews N, Araos R, Goldberg Y, et al. Duration of effectiveness of vaccines against SARS-CoV-2 infection and COVID-19 disease: results of a systematic review and meta-regression. The Lancet. 2022.
5. Barda N, Dagan N, Cohen C, Hernan MA, Lipsitch M, Kohane IS, et al. Effectiveness of a third dose of the BNT162b2 mRNA COVID-19 vaccine for preventing severe outcomes in Israel: an observational study. Lancet. 2021;398(10316):2093-100. Epub 2021/11/11.
6. https://eody.gov.gr/en/covid-19/.
7. Rubner Y, Tomasi C, Guibas LJ. The Earth Mover's Distance as a Metric for Image Retrieval. International Journal of Computer Vision. 2000;40(2):99-121.
8. https://fra.europa.eu/sites/default/files/fra_uploads/el_report_on_national_vaccine_deployment_.pdf.
9. Fiolet T, Kherabi Y, MacDonald CJ, Ghosn J, Peiffer-Smadja N. Comparing COVID-19 vaccines for their characteristics, efficacy and effectiveness against SARS-CoV-2 and variants of concern: a narrative review. Clinical microbiology and infection : the official publication of the European Society of Clinical Microbiology and Infectious Diseases. 2022;28(2):202-21. Epub 2021/10/30.
10. Harder T, Koch J, Vygen-Bonnet S, Kulper-Schiek W, Pilic A, Reda S, et al. Efficacy and effectiveness of COVID-19 vaccines against SARS-CoV-2 infection: interim results of a living systematic review, 1 January to 14 May 2021. Euro surveillance : bulletin Europeen sur les maladies transmissibles = European communicable disease bulletin. 2021;26(28). Epub 2021/07/17.
11. Singanayagam A, Hakki S, Dunning J, Madon KJ, Crone MA, Koycheva A, et al. Community transmission and viral load kinetics of the SARS-CoV-2 delta (B.1.617.2) variant in vaccinated and unvaccinated individuals in the UK: a prospective, longitudinal, cohort study. The Lancet Infectious diseases. 2022;22(2):183-95. Epub 2021/11/11.
12. Thompson MG, Stenehjem E, Grannis S, Ball SW, Naleway AL, Ong TC, et al. Effectiveness of Covid-19 Vaccines in Ambulatory and Inpatient Care Settings. The New England journal of medicine. 2021;385(15):1355-71. Epub 2021/09/09.

13. Pouwels KB, Pritchard E, Matthews PC, Stoesser N, Eyre DW, Vihta KD, et al. Effect of Delta variant on viral burden and vaccine effectiveness against new SARS-CoV-2 infections in the UK. Nature medicine. 2021;27(12):2127-35. Epub 2021/10/16.
14. Araf Y, Akter F, Tang YD, Fatemi R, Parvez MSA, Zheng C, et al. Omicron variant of SARS-CoV-2: Genomics, transmissibility, and responses to current COVID-19 vaccines. Journal of medical virology. 2022. Epub 2022/01/14.
